# Supplementary material for: Reduced oxidative capacity in macrophages results in systemic insulin resistance
Source: Nat Commun. 2018 Apr 19;9:1551. doi: 10.1038/s41467-018-03998-z (PMC5908799; doi:10.1038/s41467-018-03998-z)
Supplement: Supplementary file 1 — Supplementary Information [file 41467_2018_3998_MOESM1_ESM.pdf]

Supplementary Information

Reduced oxidative capacity in macrophages results in systemic insulin resistance

Saet-Byel Jung et al

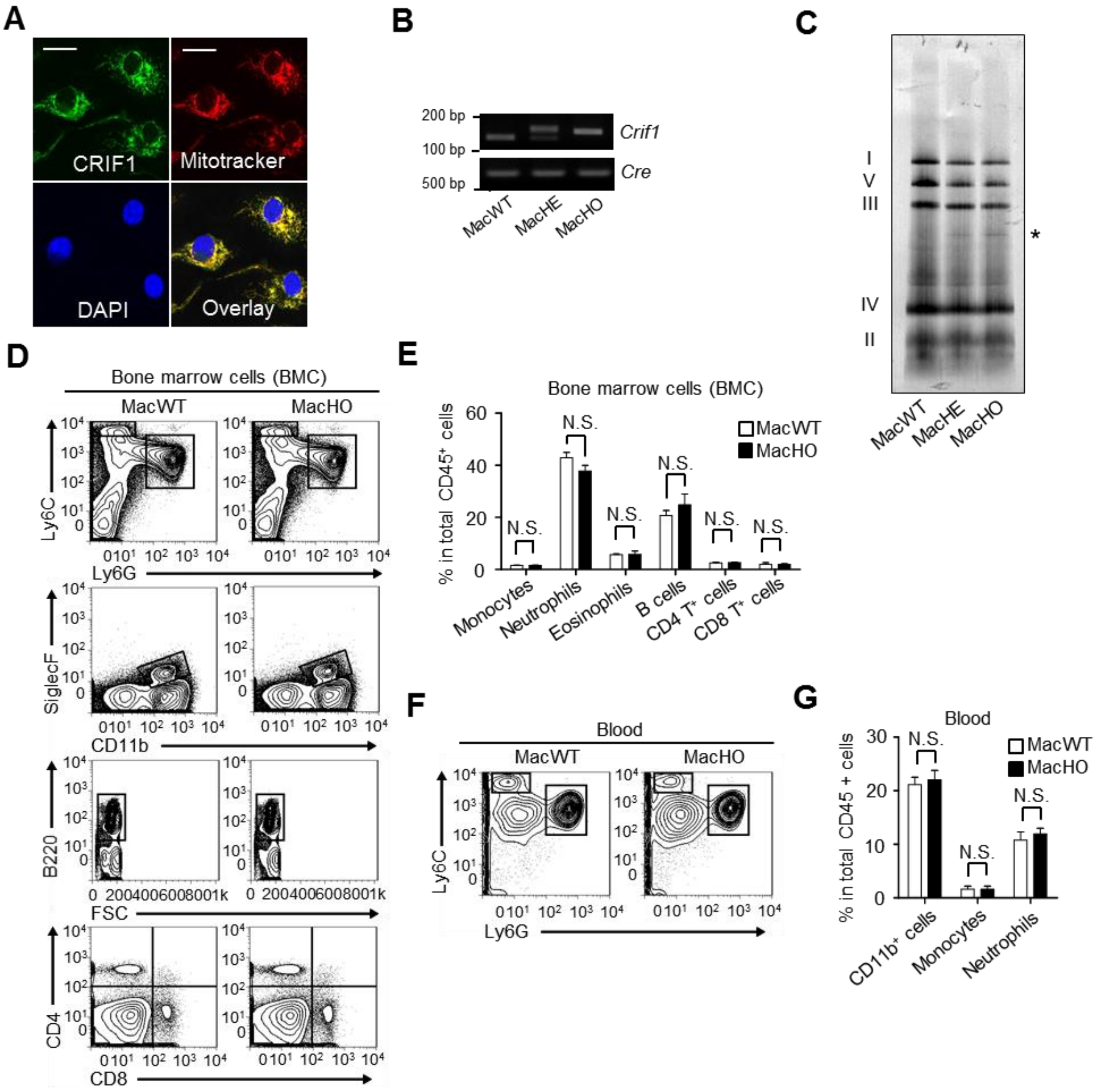

Supplementary Fig. 1. Conditional knockout of *Crif1* in mouse macrophages. (A) Immunofluorescence of endogenous CRIF1 in BMDM from 8-week-old C57BL/6J mice. BMDMs were immunostained with anti-CRIF1 antibody (green), Mitotracker (red), and DAPI (blue). (B) Genotyping to confirm mutagenesis. Primers for Cre mutant detection: LMR3066: 5'-CCC AGA AAT GCC AGA TTA CG-3', olMR3067: 5'-CTT GGG CTG CCA GAA TTT CTC-3'; primers for the flox site in Crif1 L: 5'-GGGCTGGTGAAATGTGTTG-3', R: 5'-TCAGCTAGGGTGGGACAGA-3'. (C) BN-PAGE analysis of OxPhos complexes. Mitochondria were isolated from BMDMs to assess the steady-state levels of OxPhos complexes. *, abnormal sub-complexes. (D and E) FACS analysis in bone marrow cells from 8-week-old MacWT and MacHO mice. Bone marrow cells were stained for monocytes ($CD45^{+}$/$Ly6G^{low}$/$Ly6C^{high}$), neutrophils ($CD45^{+}$/$Ly6G^{high}$/$Ly6C^{low}$), eosinophils ($CD45^{+}$/$CD11b^{+}$/$SiglecF^{+}$), B cells ($CD45^{+}$/$B220^{+}$), $CD4^{+}$ T cells ($CD45^{+}$/CD/3^{+}/$CD4^{+}$), and $CD8^{+}$ T ($CD45^{+}$/$CD3^{+}$/$CD8^{+}$) cells. N.S., not significant. (F and G) FACS analysis in blood cells from 8-week-old MacWT and MacHO mice . Blood cells were obtained using RBC lysis buffer and stained for monocytes ($CD45^{+}$, $Ly6G^{low}$, $Ly6C^{high}$) and neutrophils ($CD45^{+}$, $Ly6G^{high}$, $Ly6C^{low}$). Data are representative of three independent experiments (n=5 mice per group).

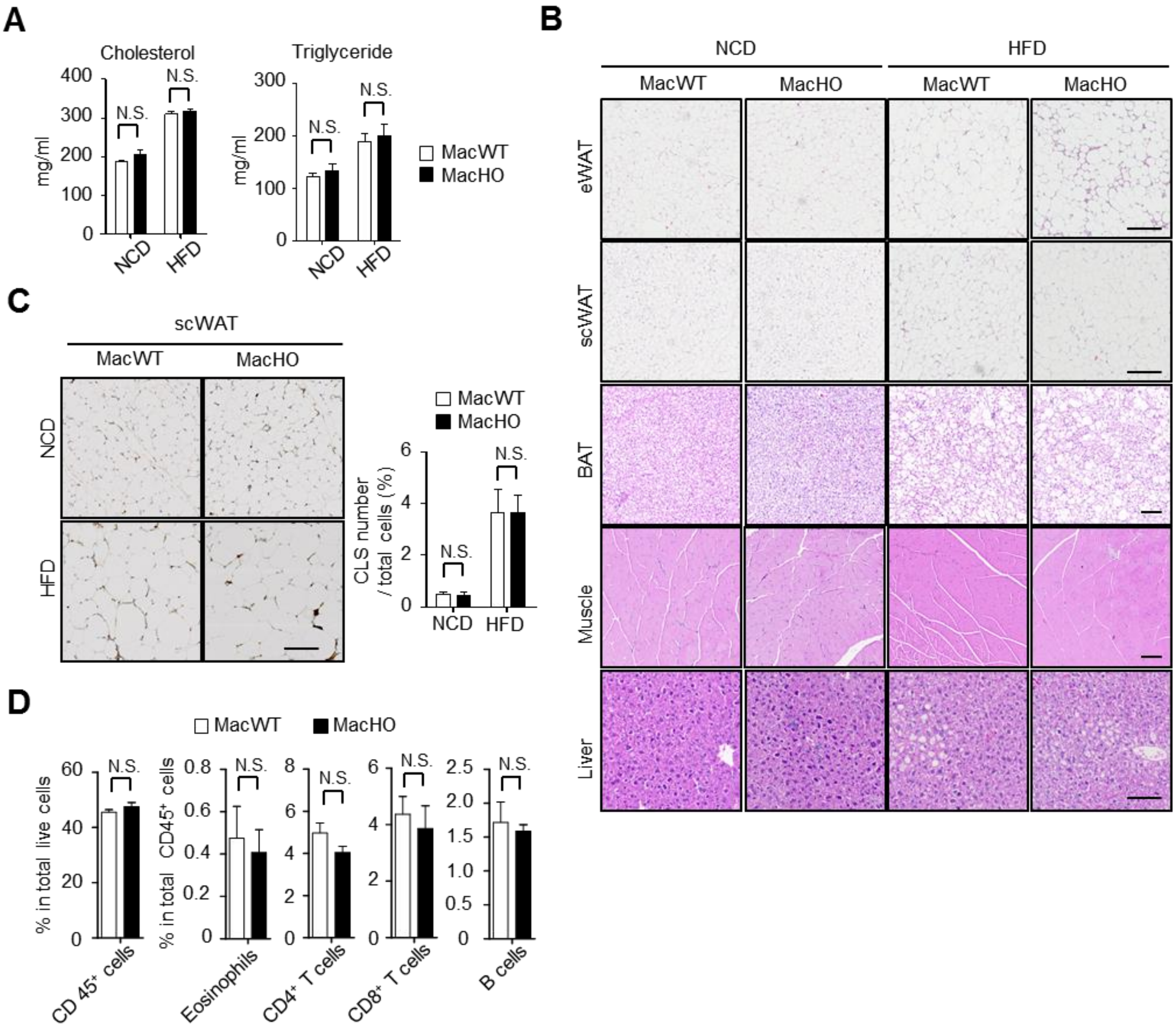

Supplementary Fig. 2. HFD affects the macrophage population in eWAT and scWAT independent of CRIF1 deficiency. (A) Cholesterol and triglyceride level in blood from MacWT and MacHO mice after NCD and HFD (n=4 per group). (B) H&E staining of eWAT, scWAT, BAT, muscle, and liver in MacWT or MacHO mice after NCD and HFD. Scale bar: 200 µm. (C) Immunohistochemistry with anti-F4/80 and hematoxylin staining to detect macrophage accumulation in scWAT in 8 week HFD-fed MacWT or MacHO mice. Scale bar: 200 µm. (D) FACS analysis of infiltrated immune cells ($CD45^+$) in eWAT, and percentages of eosinophils ($CD45^+/CD11b^+/SiglecF^+$), $CD4^+$ T cells ($CD45^+/CD3^+/CD4^+$), $CD8^+$ T cells ($CD45^+/CD3^+/CD8^+$), and B cells ($CD45^+/B220^+$) after HFD. (E) Representative FACS strategy for macrophages using SVCs from eWAT in 8 week HFD-fed MacWT or MacHO mice. Data represent means ± SEM and are representative of three independent (n=5 mice per group).

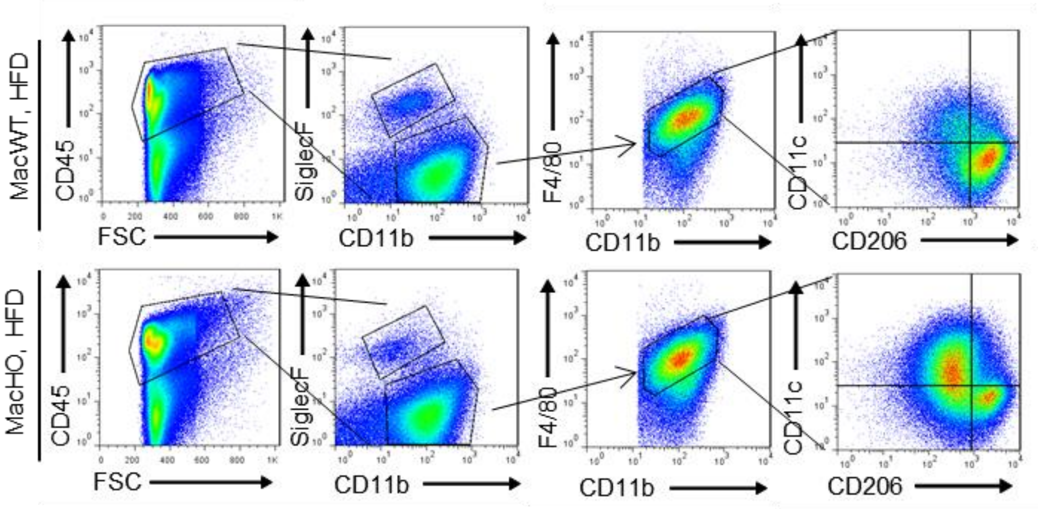

Supplementary Fig. 3. FACS strategy for macrophages using SVCs. FACS gating of total ($CD45^+$/$F4/80^+$/$CD11b^+$), M1 ($CD11c^+$/$CD206^-$) and M2 ($CD11c^-$/$CD206^+$) macrophages infiltrated into eWAT of 8 week HFD-fed MacWT or MacHO mice in figure 2j.

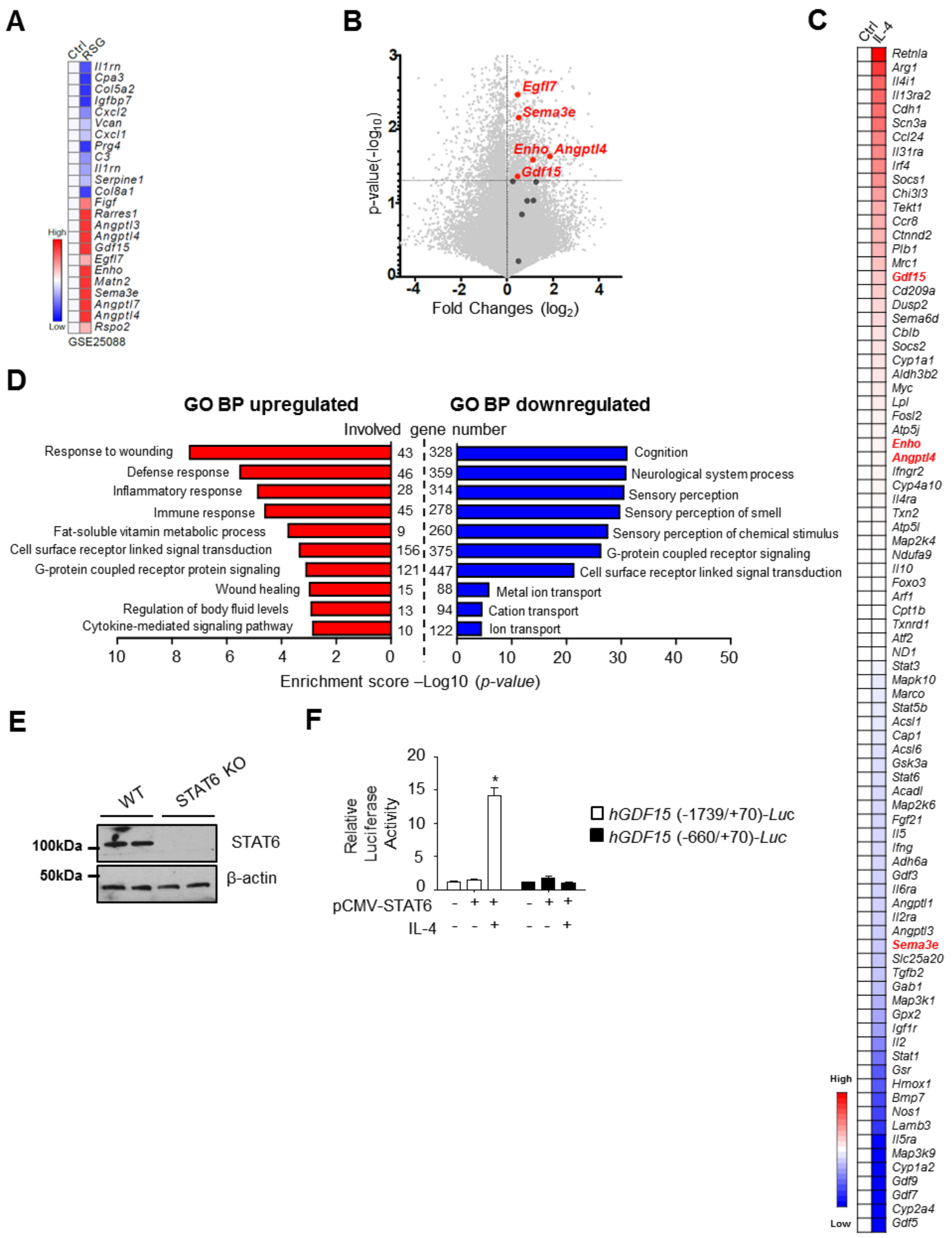

Supplementary Fig. 4. IL-4 and RSG induced GDF15 through STAT6 activation. (A) Heat map showing expression of humoral factors upregulated/downregulated in rosiglitazone (RSG)-treated human macrophages (GSE25088). (B) Volcano plot showing RSG-induced genes in human macrophages. (C) Heat map showing expression of various upregulated or downregulated genes in IL-4–treated murine macrophages. (D) mRNAs induced by IL-4 in murine macrophages (from panel C) were functionally classified by GO analysis. (E) Immunoblot analysis of STAT6 in WT and *Stat6*-KO BMDMs. (F) Relative luciferase activities of human *GDF15* promoter in RAW 264.7 cells transfected with *hGDF15* (-1739/+70)-*Luc* or *hGDF15* (-660/+70)-*Luc* with pCMV-STAT6 vector and cultured with IL-4 (100 ng/ml) for 24 h. Data represent means ± SEM and are representative of three independent experiments. *$p<0.05$ (two-tailed Student's *t* test).

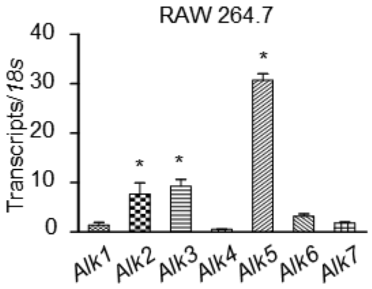

Supplementary Fig. 5. ALK expression in murine macrophages. Real-time PCR analysis of TGF-β RI (ALK1–7) expression in RAW 264.7 cells. Data represent means ± SEM and are representative of three independent experiments. *p<0.05 (two-tailed Student's *t* test).

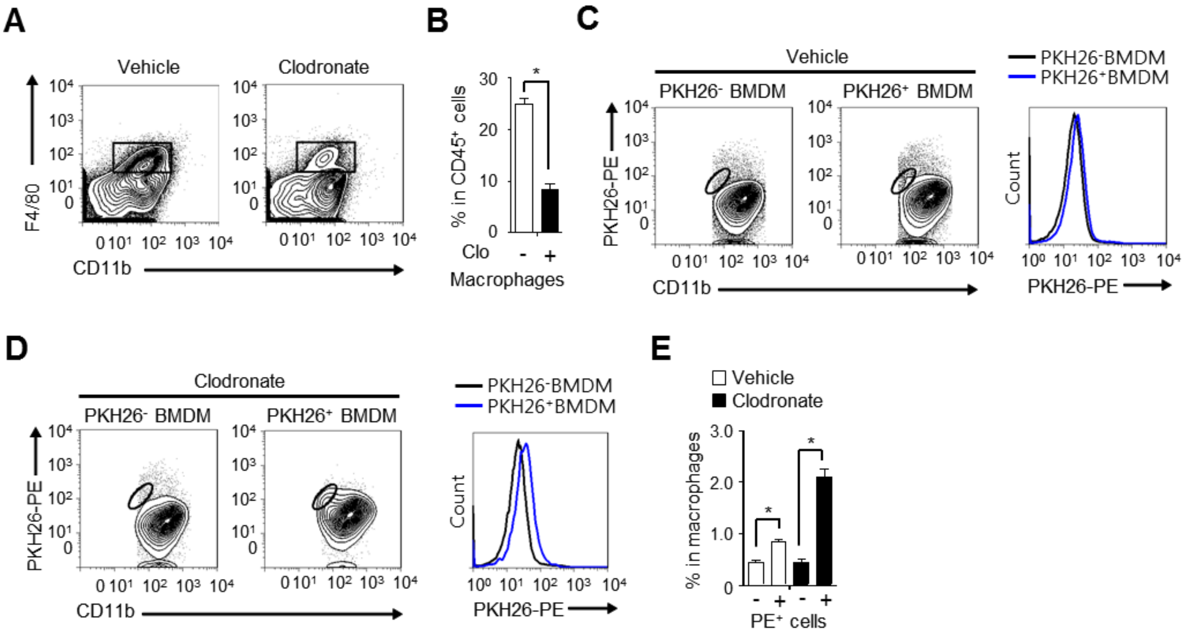

Supplementary Fig. 6. Depletion and reconstitution of macrophages in epididymal adipose tissue by clodronate treatment. (A and B) Reduced macrophage populations in SVF of eWAT after intraperitoneal injection of clodronate liposomes. (C–E) FACS analysis of percentage of infiltrated PKH^{+} macrophages in eWAT after two intravenous injections of BMDMs with or without clodronate liposomes. Data represent means ± SEM and are representative of three independent experiments (n=5 mice per group). *p<0.05 (two-tailed Student's t test).

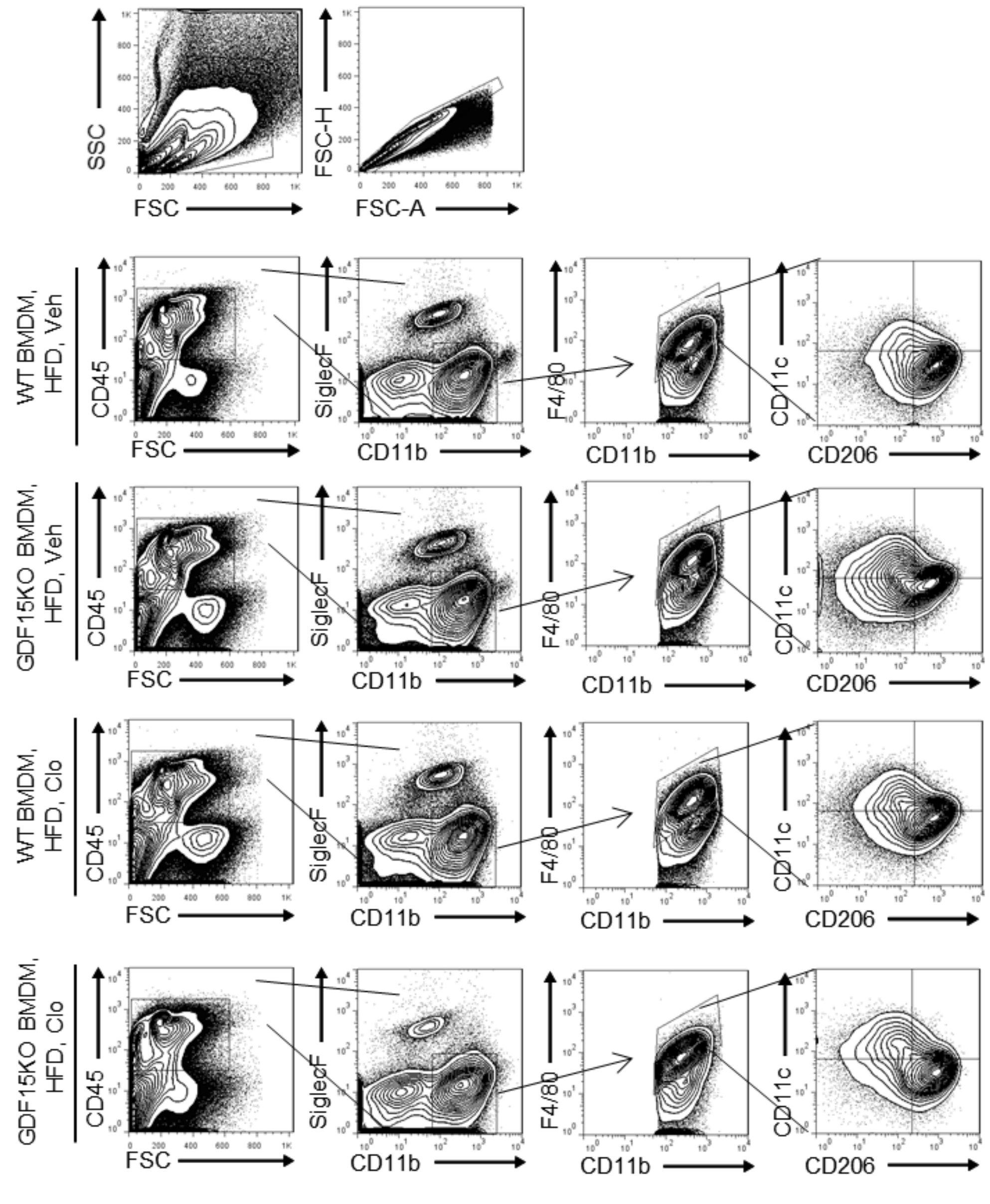

Supplementary Fig. 7. FACS gating strategy for macrophages using SVCs. FACS gating of total (CD45^{+}/F4/80^{+}/CD11b^{+}), M1 (CD11c^{+}/CD206^{-}) and M2 (CD11c^{-}/CD206^{+}) macrophages infiltrated into eWAT of WT or GDF15KO BMDM injected into mice after 8.5 weeks of HFD for figure 5j, 5k and 5l.

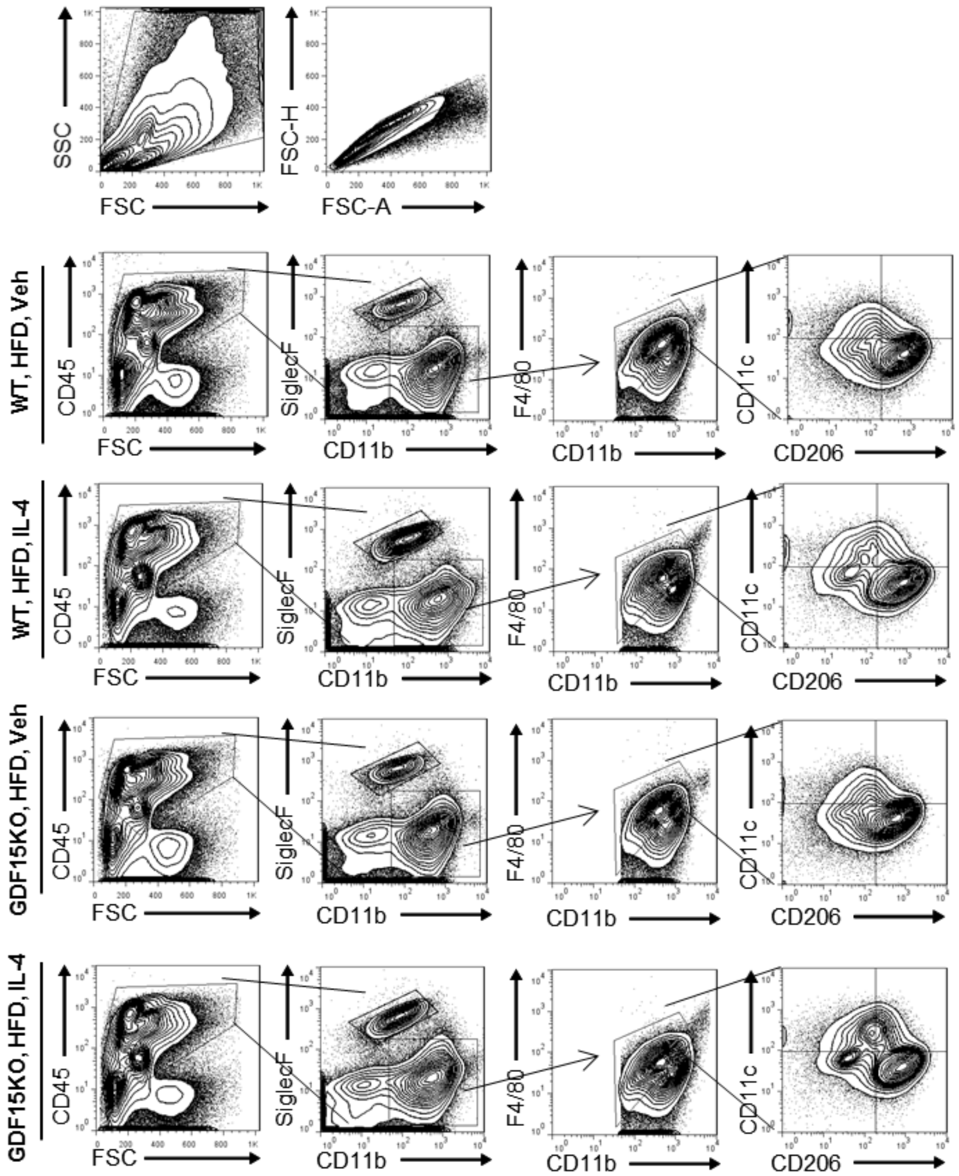

Supplementary Fig. 8. FACS strategy for macrophages using SVCs. FACS gating of total ($CD45^+/F4/80^+/CD11b^+$), M1 ($CD11c^+/CD206^-$) and M2 ($CD11c^-/CD206^+$) macrophages infiltrated into eWAT of WT or GDF15KO mice with or without IL-4 IP injection after 7 weeks of HFD for figure 6f, 6g, 6h and 6i.

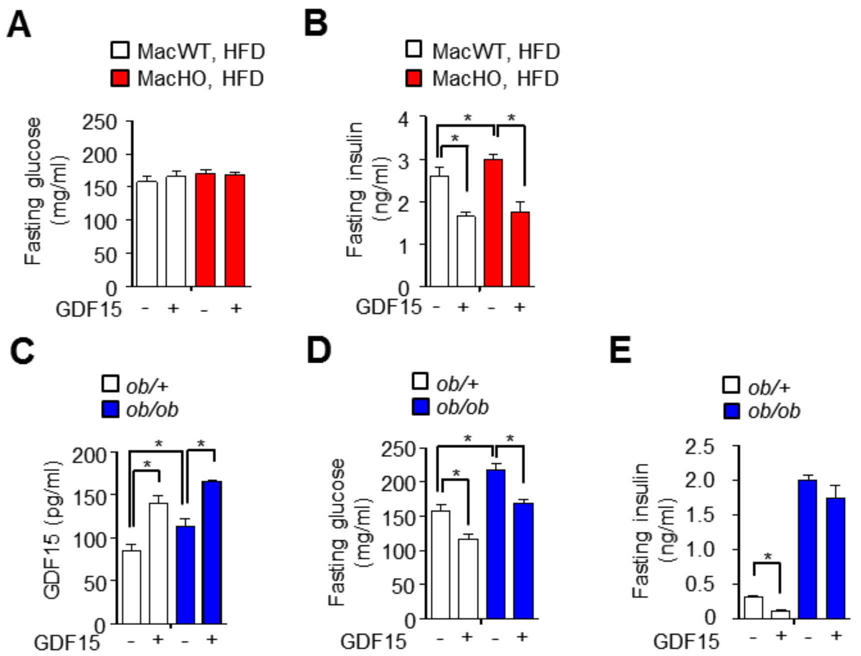

Supplementary Fig. 9. GDF15 reverses insulin resistance caused by reduced oxidative function in macrophages. (A) Glucose levels of 12 week HFD-fed MacWT or MacHO mice after intraperitoneal injection of rGDF15 for 2 weeks. HFD-fed MacWT or MacHO mice were injected intraperitoneally with rGDF15 (300 μg/kg) or vehicle for 2 weeks every other day (n=5 per group). (B) Fasting insulin levels of 12 week HFD-fed MacWT or MacHO mice after intraperitoneal injection of rGDF15 for 2 weeks. (C) Serum GDF15 levels of *ob/+* and *ob/ob* mice after intraperitoneal injection of rGDF15 (300 μg/kg) for 2 weeks (n=5 per group). (D) Fasting glucose levels of *ob/+* and *ob/ob* mice after intraperitoneal injection of rGDF15 for 2 weeks. (E) Fasting insulin levels of *ob/+* and *ob/ob* mice after intraperitoneal injection of rGDF15 for 2 weeks. Data represent means ± SEM and are representative of three independent experiments (n=5 mice per group). *p<0.05 (two-tailed Student's *t* test).

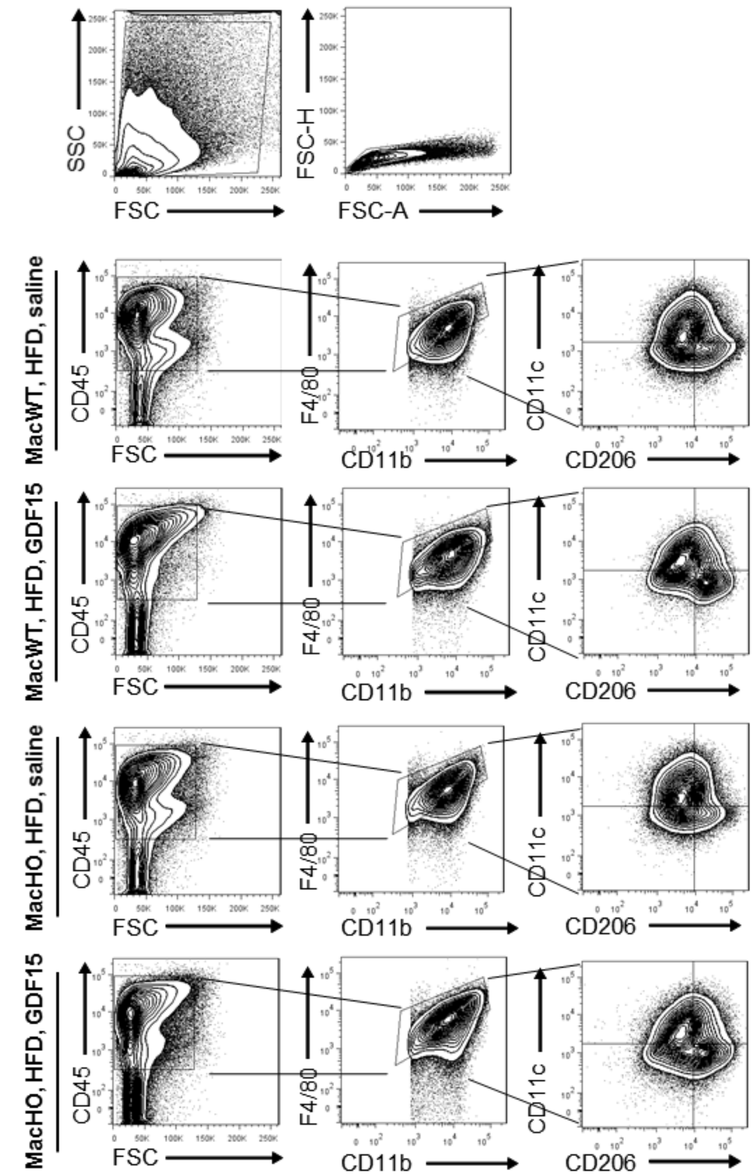

Supplementary Fig. 10. FACS strategy for macrophages using SVCs. FACS gating of total ($CD45^+/F4/80^+/CD11b^+$), M1 ($CD11c^+/CD206^-$) and M2 ($CD11c^-/CD206^+$) macrophages infiltrated into eWAT of MacWT or MacHO mice with or without GDF15 IP injection after 8 weeks of HFD for figure 8d and 8e.

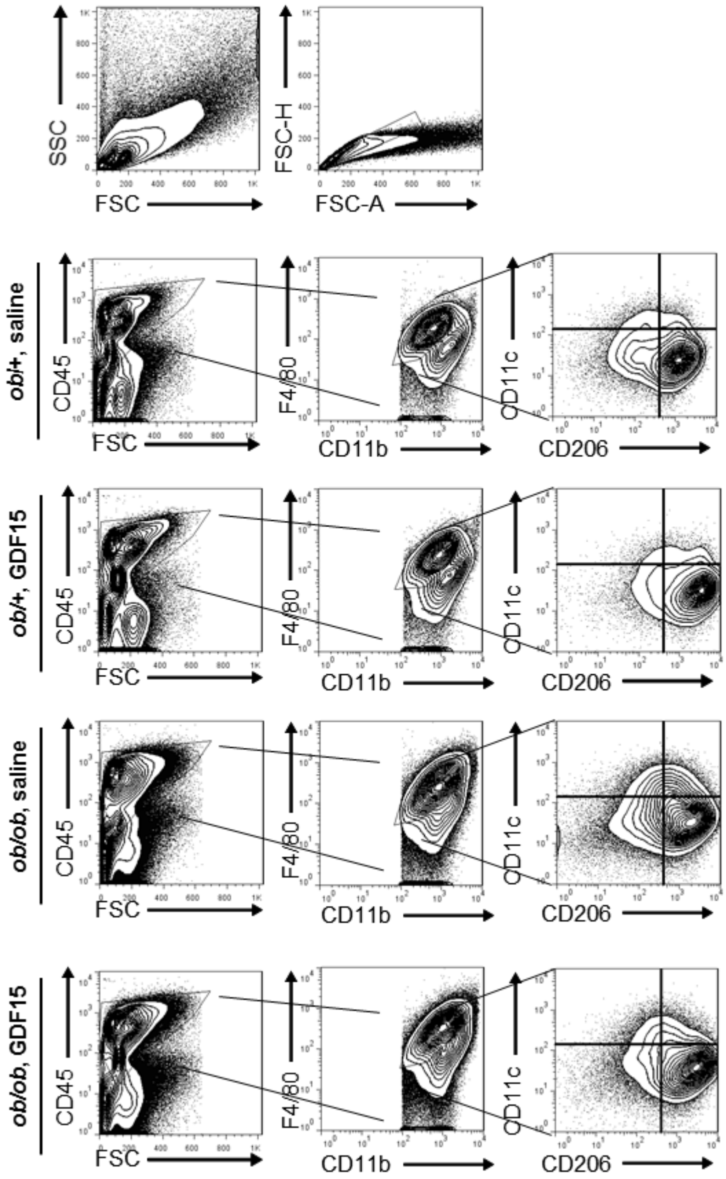

Supplementary Fig. 11. FACS strategy for macrophages using SVCs. FACS gating of total ($CD45^+$/$F4/80^+$/$CD11b^+$), M1 ($CD11c^+$/$CD206^-$) and M2 ($CD11c^-$/$CD206^+$) macrophages infiltrated into eWAT of *ob/*+ or *ob/ob* mice with or without GDF15 IP injection for figure 8h and 8i.

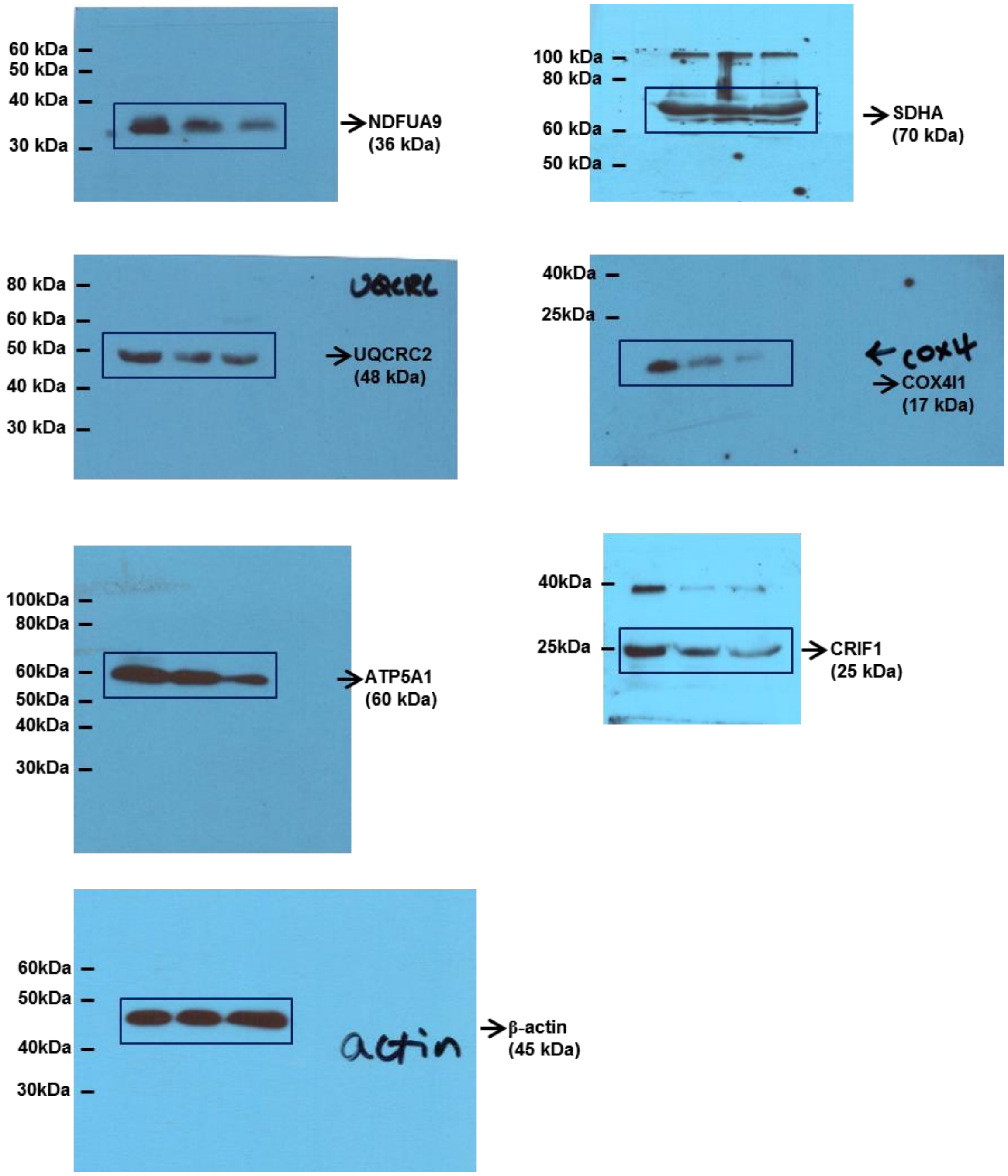

Supplementary Fig. 12. Uncropped images of western blot presented in figure 1b. Blue boxes show the region cropped from each blot and presented in figure 1b.

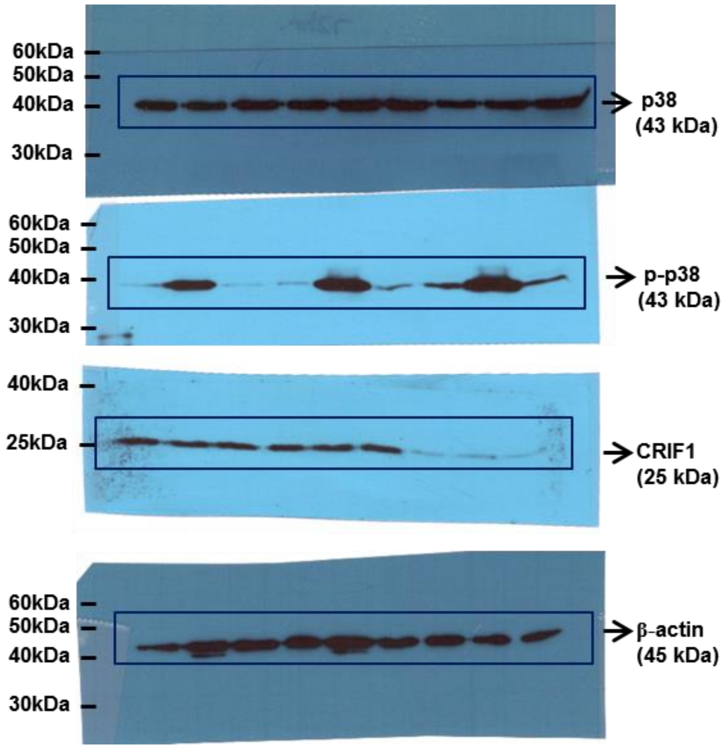

Supplementary Fig. 13. Uncropped images of western blot presented in figure 1k. Blue boxes show the region cropped from each blot and presented in figure 1k.

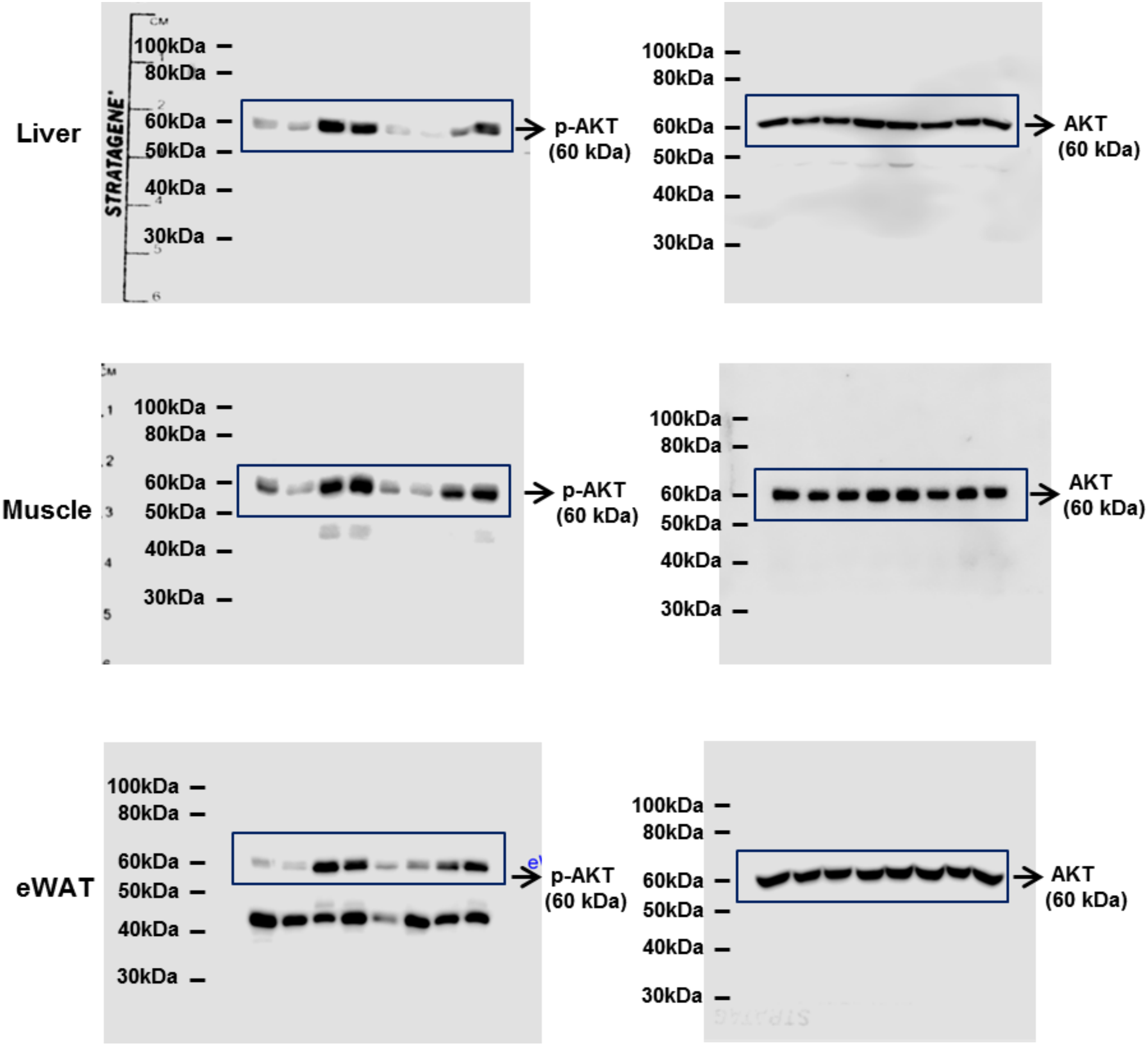

Supplementary Fig. 14. Uncropped images of western blot presented in figure 2d. Blue boxes show the region cropped from each blot and presented in figure 2d.

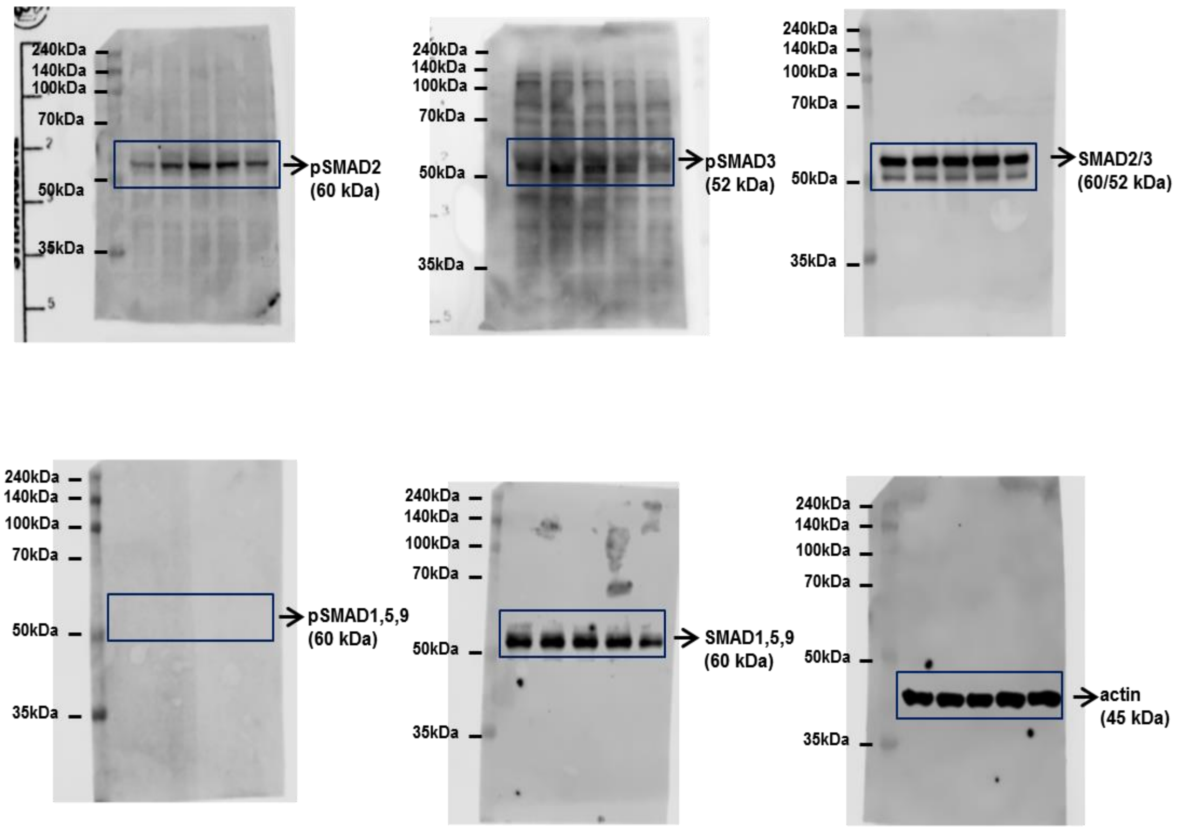

Supplementary Fig. 15. Uncropped images of western blot presented in figure 4b. Blue boxes show the region cropped from each blot and presented in figure 4b.

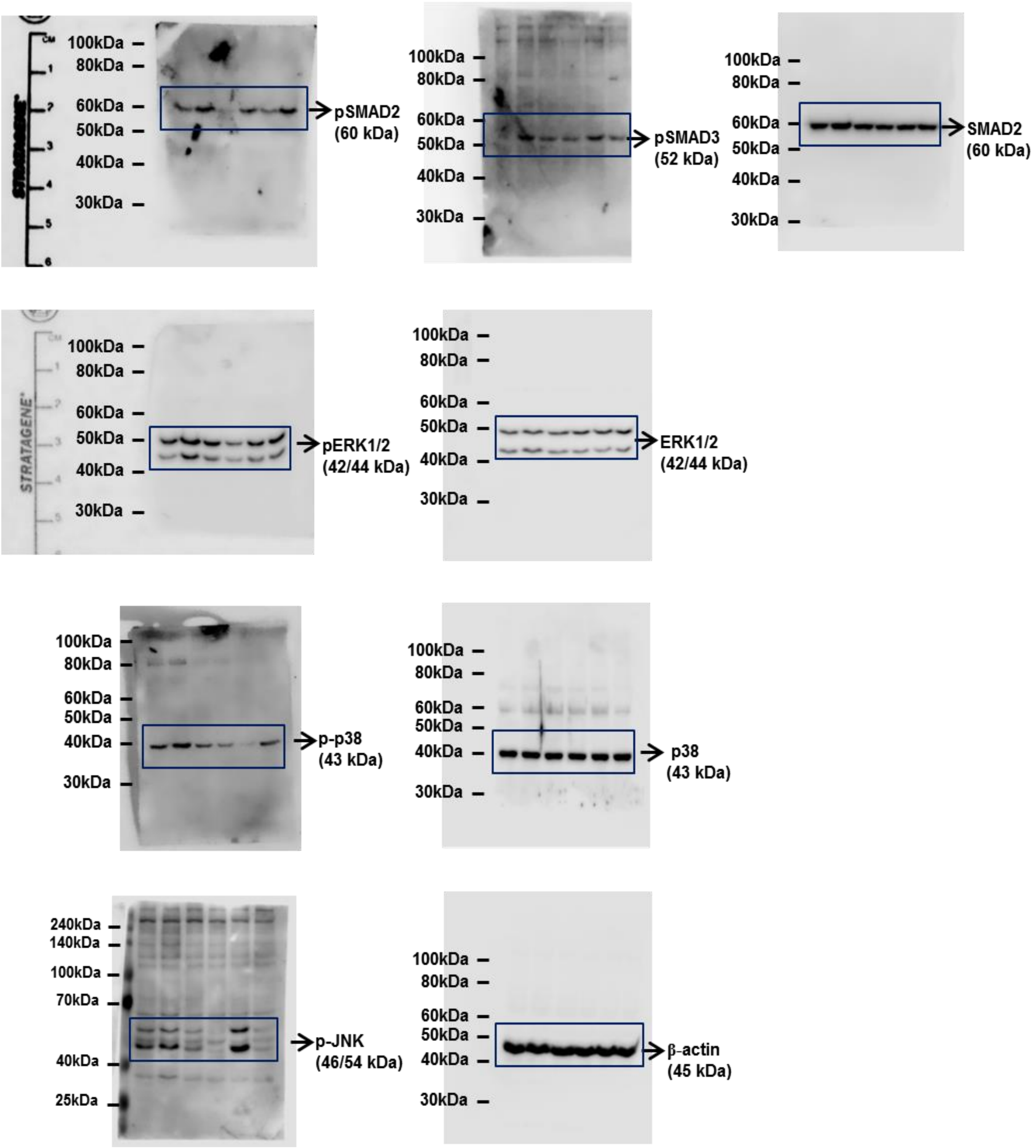

Supplementary Fig. 16. Uncropped images of western blot presented in figure 4c. Blue boxes show the region cropped from each blot and presented in figure 4c.

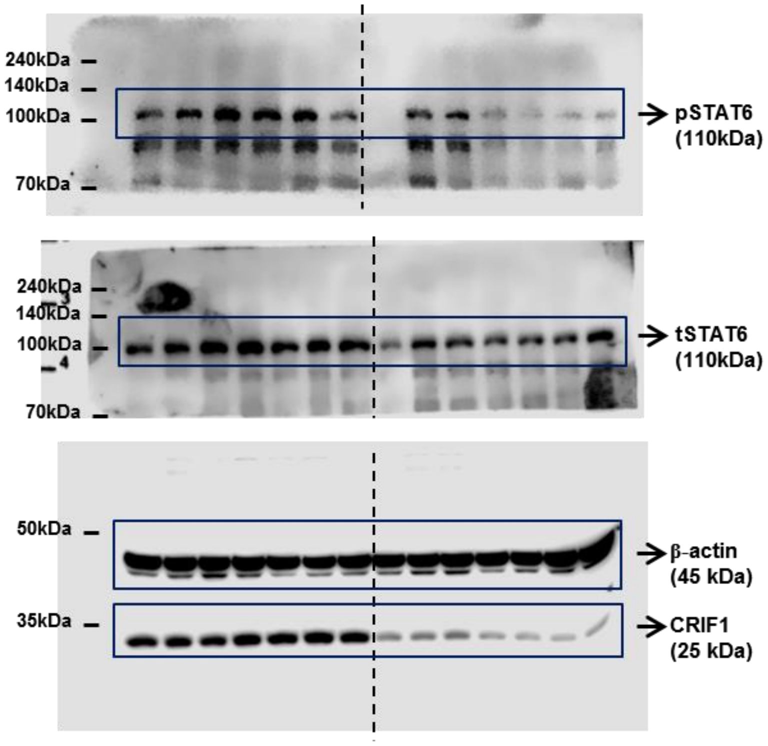

Supplementary Fig. 17. Uncropped images of western blot presented in figure 7b. Blue boxes show the region cropped from each blot and presented in figure 7b.

Supplementary Table 1. Primer pairs used for qRT-PCR

Gene	Forward	Reverse
18s	5′-CTGGTTGATCCTGCCAGTAG-3′	5′-CGACCAAAGGAACCATAACT-3′
Angptl4	5′-TCA AAGACTCCGAGGATAGA-3′	5′-AAAGCCCTTTTCGTAGTTTT-3′
Gdf15	5′-GAGCTACGGGGTCGCTTC-3′	5′-GGGACCCCAATCTCACCT-3′
Enho	5′-CTCATCGCCATCGTCTGCAAT-3′	5′-CGCACTGGATTCCGAGAGAGA-3′
Egfl7	5′-TGCGACGGACACAGAGCCTGCA-3′	5′-CGCACTGGATTCCGAGAGAGA-3′
Sema3e	5′-GACGGCTACAGAGAGATATACTGGC-3′	5′-AACAGGGGTTCCTCTGAATGG-3′
Stat6	5′-TCTCCACGAGCTTCACATTG-3′	5′-CTTGGTTACCATGCACGATG-3′
Alk1	5′-TTGGTGCAGAGGACGGTAG-3′	5′-TGCTCATCTCGTGAGGAGAAAAT-3′
Alk2	5′-TTTAAGAGACGCAATCAAGAGCG-3′	5′-TCCAACAGGGTTATCTGGCGA-3′
Alk3	5′-TGCAAGGATTCACCGAAAGC-3′	5′-TGCCATCAAAGAACGGACCTAT-3′
Alk4	5′-CGTGTCTACCATAACCGCCAG-3′	5′-GCCCTTGCCGATAATCTCTTGTA-3′
Alk5	5′-AAAACAGGGGCAGTTACTACAAC-3′	5′-TGGCAGATATAGACCATCAGCA-3′
Alk6	5′-CCGACCTCGGTACAGCATTG-3′	5′-GCTCTGAGACTGCTCGATCAAG-3′
Alk7	5′-ATGCTAACCAACGGGAAAGAG-3′	5′-GGAAGGTGCAGTGTGATATTGT-3′
Crif1	5′-GAACGCTGGGAGAAAATTCA-3′	5′-CTCCGATTATTAGTGGGACA-3′
Cpt1a	5′-TATAACAGGTGGTTTGAC-3′	5′-CAGAGGTGCCCAATGATG-3′
Acadm	5′-TGACGGAGCAGCCAATGA-3′	5′-TCGTCACCCTTCTTCTCTGCTT-3′
Acadvl	5′-TTACATGCTGAGTGCCAACATG-3′	5′-CGCCTCCGAGCAAAAGATT-3′
AcoOx	5′-ATGACCCCACTTCCTGACAC-3′	5′-GAAGGTCAGCCACCATGATT-3′
Pgc1a	5′-TCACACCAAACCCACAGAAA-3′	5′-CTTGGGGTCATTTGGTGACT-3′
Il-6	5′-CCGGAGAGGAGACTTCACAG-3′	5′-CAGAATTGCCATTGCACAAC-3′
iNOS	5′-CACCTTGGAGTTCACCCAGT-3′	5′-ACCACTACTCGTACTTGGGATGC-3′
Mcp1	5′-CCCAATGAGTAGGCTGGAGA-3′	5′-TCTGGACCCATTCCTTCTTG-3′
Tnfa	5′-CCCCAAAGGGATGAGAAGTT-3′	5′-CACTTGGTGGTTTGCTACGA-3′
Arg1	5′-ATGGAAGAGACCTTCAGCTAC-3′	5′-GCTGTCTTCCCAAGAGTTGGG-3′
Ym1	5′-GGGCATACCTTTATCCTGAG-3′	5′-CCACTGAAGTCATCCATGTC-3′
Pparg	5′-ATCTTAACTGCCGGATCCAC-3′	5′-TGGTGATTTGTCCGTTGTCT-3′
Il-10	5′-TGCACTACCAAAGCCACAAG-3′	5′-TAAGAGCAGGCAGCATAGCA-3′
IL-4Ra	5′-AGGCCCCAGTACAGAATGTG-3′	5′-TCTCAGGTGACATGCTCAGG-3′
Fizz1	5′-CCTTCTCATCTGCATCTCCCTG-3′	5′-GCTGGATTGGCAAGAAGTTCC-3′

Supplementary Table 2. Antibodies used for FACS analysis

Antibody	Fluorochrome	Clone	Supplier	Cat no.
Fc block		2.4G2	BD Bioscience	553142
BD Pharmingen, 7AAD	PerCP-Cy5.5		BD Bioscience	51-68981E
CD45.2	PE	104	BD Bioscience	560695
CD45.2	PE/Cy7	104	Biolegend	109830
CD45.2	APC/Cy7	104	Biolegend	109824
CD45.2	APC	104	Biolegend	109814
CD45	Alexa 700	30-F11	Biolegend	103128
CD4	PerCP-Cy5.5	GK1.5	Biolegend	100434
CD4	PE	GK1.5	Biolegend	100407
CD4	APC	GK1.5	Biolegend	100412
CD4	FITC	GK1.5	Biolegend	100405
CD8a	PE	53-6.7	Biolegend	100708
CD8a	FITC	53-6.7	Biolegend	100706
CD3	APC/Cy7	145-2C11	Biolegend	100330
CD3	PE	145-2C11	Biolegend	100308
CD206	APC	C068C2	Biolegend	141708
F4/80	FITC	BM8	Biolegend	123108
F4/80	PE	BM8	Biolegend	123110
F4/80	APC	BM8	Biolegend	123116
CD11c	PE	N418	eBioscience	12-0114-81
CD45R/B220	APC/Cy7	RA3-6B2	Biolegend	103223
CD45R/B220	FITC	RA3-6B2	Biolegend	103206
Ly6G	FITC	1A8	Biolegend	127605
Ly6C	PE	HK1.4	Biolegend	128007
CD11b	PerCP-Cy5.5	M1/70	BD Bioscience	550993
CD11b	PE	M1/70	Biolegend	557397
CD11b	PE/Cy7	M1/70	Biolegend	101216
CD11b	APC/Cy7	M1/70	Biolegend	101226
CD11b	APC	M1/70	BD Bioscience	553312
CD11b	FITC	M1/70	BD Bioscience	553310
SiglecF	PE	E50-2440	BD Bioscience	552128
