# Supplementary material for: Reduced oxidative capacity in macrophages results in systemic insulin resistance
Source: Nat Commun. 2018 Apr 19;9:1551. doi: 10.1038/s41467-018-03998-z (PMC5908799; doi:10.1038/s41467-018-03998-z)
Supplement: Supplementary file 3 — Description of Additional Supplementary Files [file 41467_2018_3998_MOESM3_ESM.pdf]

Description of Additional Supplementary Files

File Name: Supplementary Data 1
Description: Supplementary data 1 is a list of fold changes ($p < 0.05$, Cutoff: Log Fold change ≥ 1 or ≤ -1).

File Name: Supplementary Data 2
Description: Gene ontology (GO) biological process analysis data using the genes with a log fold change ≥ 1 or ≤ -1.

File Name: Supplementary Data 3
Description: Gene ontology (GO) biological process analysis data using the genes with a log fold change ≥ 1 or ≤ -1.
